# Supplementary figures and images for: A novel stochastic simulation approach enables exploration of mechanisms for regulating polarity site movement
Source: PLoS Comput Biol. 2021 Jul 15;17(7):e1008525. doi: 10.1371/journal.pcbi.1008525 (PMC8315557; doi:10.1371/journal.pcbi.1008525)

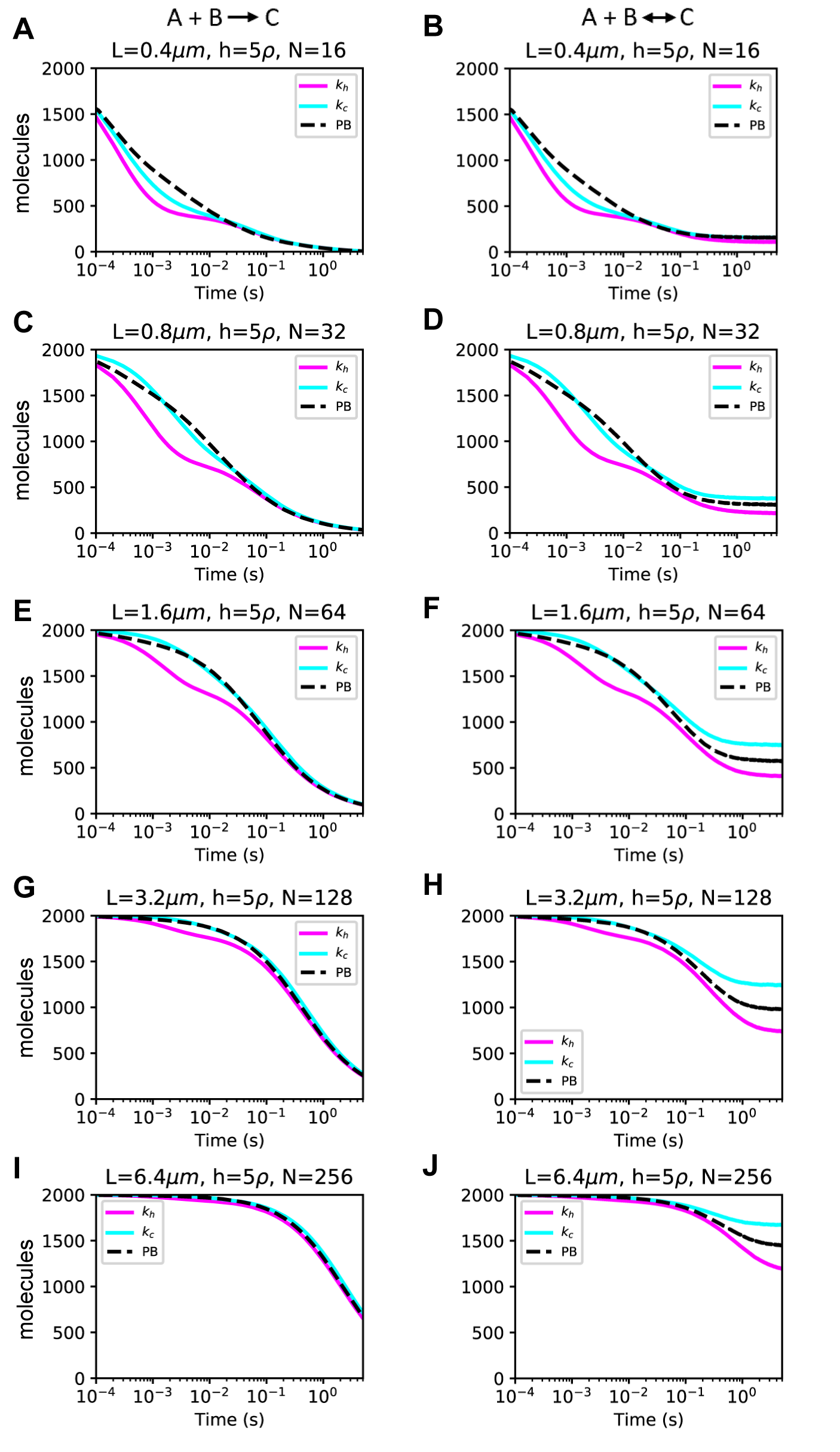

Supplement: S1 Fig — We present the mean total number of species A as a function of time. The domain sizes are L = 0.4 μm (A, B), 0.8 μm (C, D), 1.6 μm (E, F), 3.2 μm (G, H) and 6.4 μm (I, K). Initial molecule abundances are A = B = 2000, C = 0. In all the simulations, the degree of diffusion control is λπρ2/Dtot = 50, with Dtot = 2D and D = 0.0025μm2/s, ρ = 0.005 μm and λ = 3183.1/s, with Dtot = 2D and D = 0.0025μm2/s. h = 5ρ and the number of grid elements is N2 = (L/h)2. For the reversible reaction (B, D, F, H, K), the microscopic dissociation rate constant kdmicro is 10/s. (TIF) [file pcbi.1008525.s001.tif]

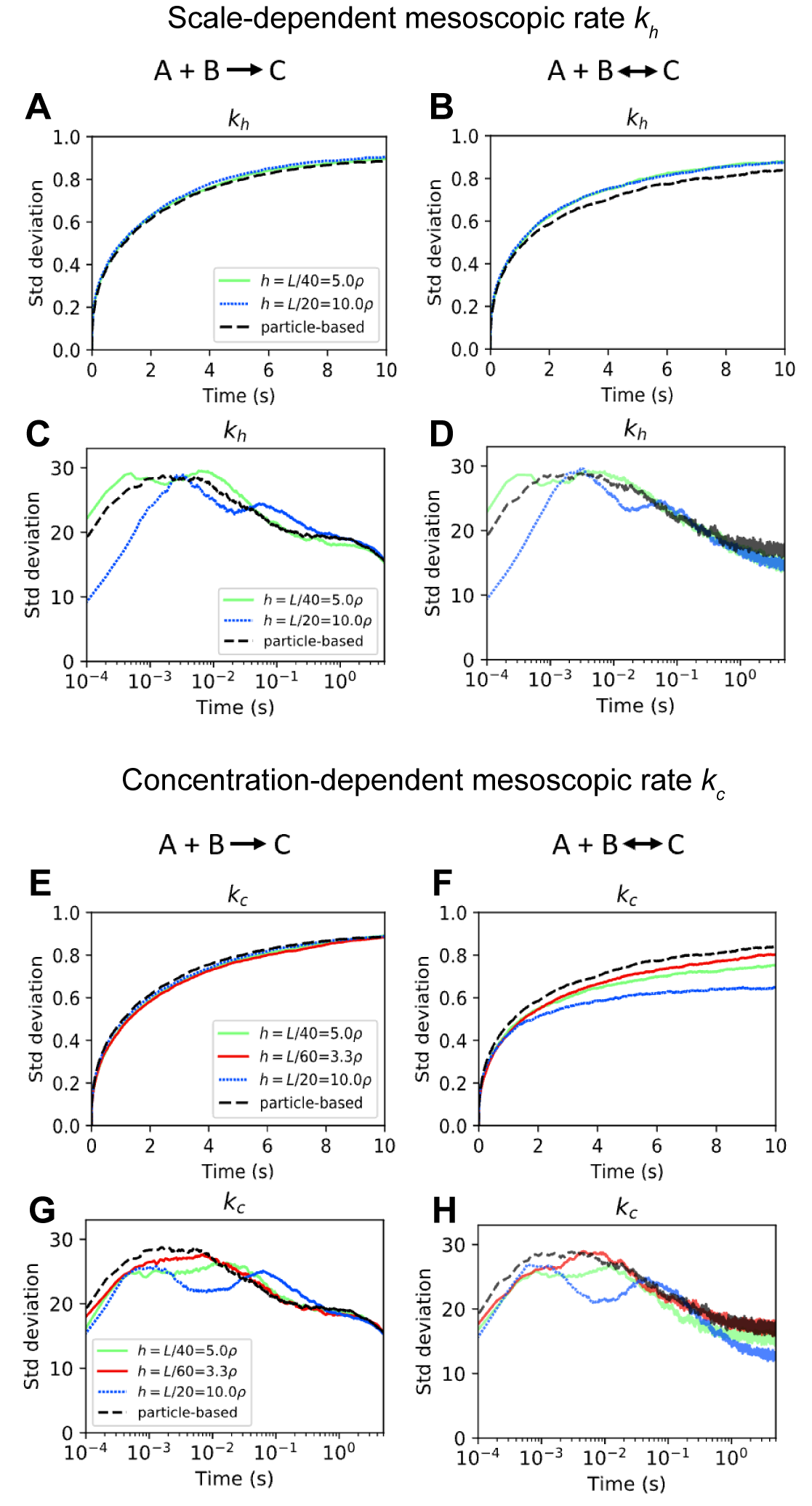

Supplement: S2 Fig — The mean as a function of time is shown in Fig 2 of the main text. We present the standard deviation of total number of species A as a function of time. (A-D) show spatial Gillespie simulations using the mesoscopic rate kh with initial low abundance of reactants in (A,B) (total A = total B = 5, total C = 0 at t = 0) and initial high abundance (total A = total B = 5000, total C = 0 at t = 0) in (C-D). (E-H) show corresponding simulations to (A-D) but using the mesoscopic rate kc. In all the simulations, the degree of diffusion control is λπρ2/Dtot = 50, with Dtot = 2D and D = 0.0025μm2/s, ρ = 0.005 μm and λ = 3183.1/s. The size of the domain is L = 1μm. For the reversible reaction, the microscopic dissociation rate constant kdmicro is 1/s in panels (B, F), and 10/s in panels (D, H). (TIF) [file pcbi.1008525.s002.tif]

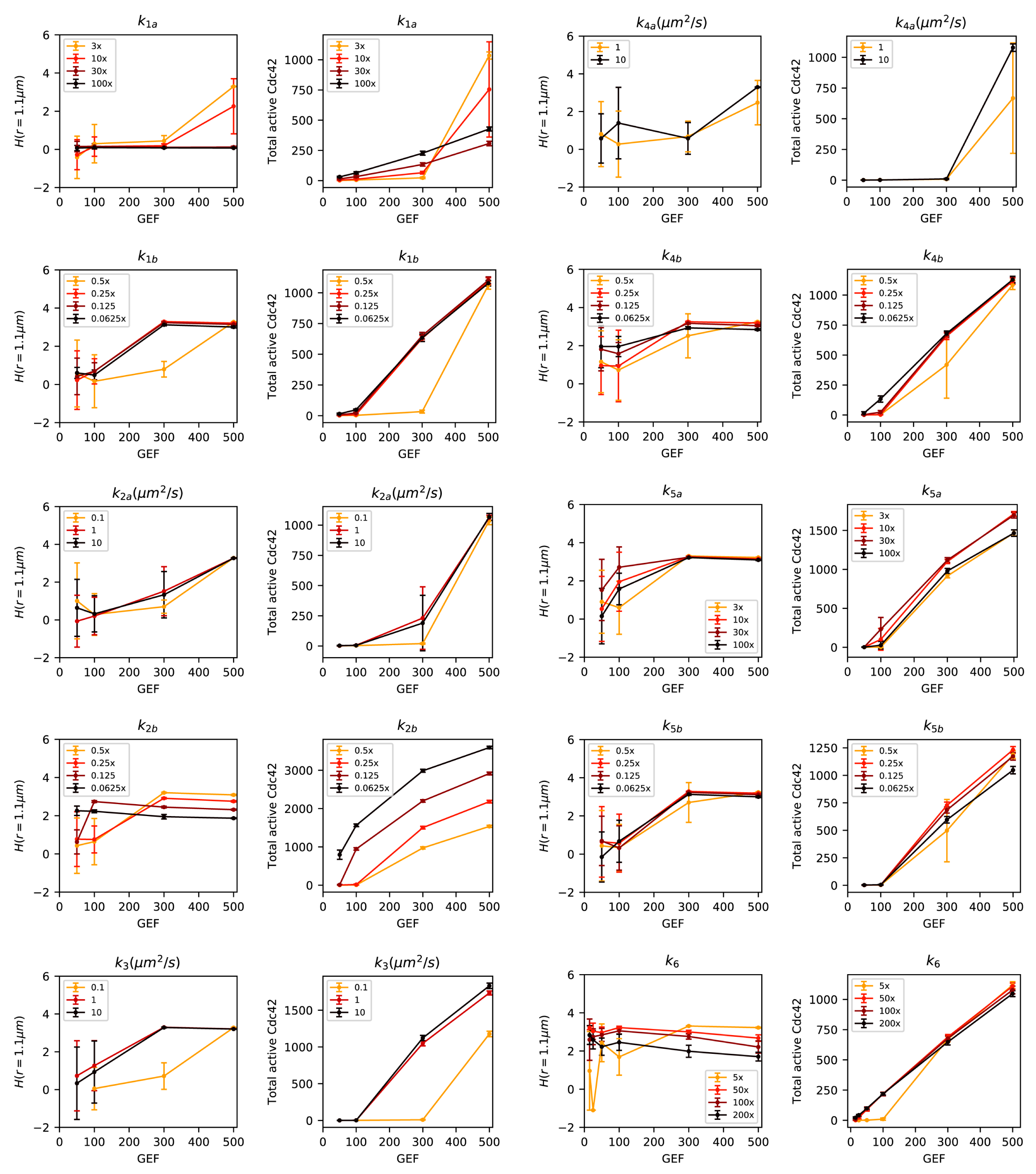

Supplement: S3 Fig — In each panel the rate constant in the title of the figure is varied as indicated in the legend. For each parameter set, the simulations were initialized with an unpolarized random distribution with all GEF and Cdc42 in the cytosol and 10 min were simulated to provide enough time for the system to polarize. Mean and standard deviation (error bars) were calculated sampling every 30s for the last 5min of each simulation with data from 3 independent simulations. (TIF) [file pcbi.1008525.s003.tif]

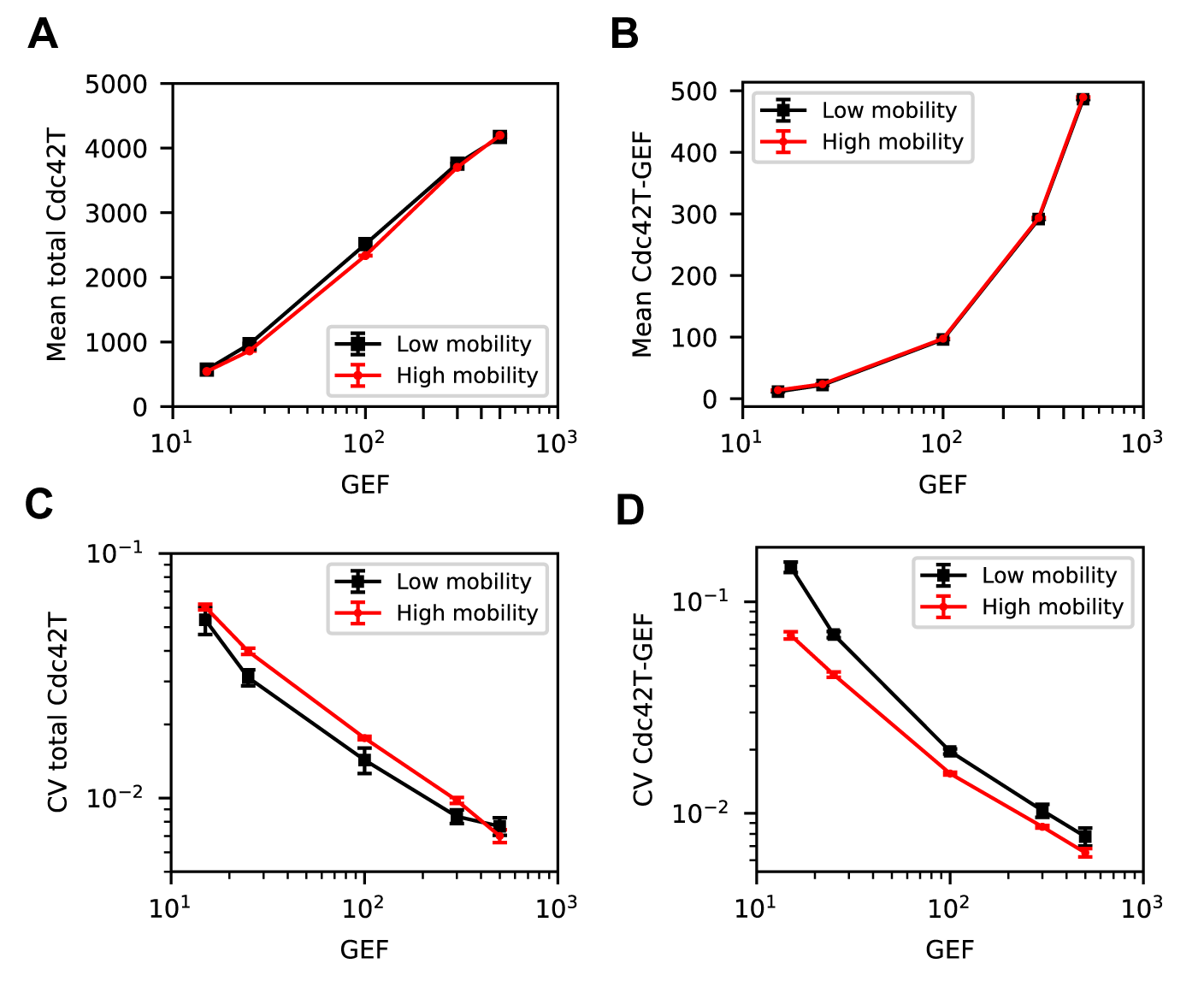

Supplement: S4 Fig — (A) Mean number of total Cdc42T, (B) coefficient of variation (CV) of total Cdc42T, (C) mean number of Cdc42T-GEF, (D) CV of Cdc42T-GEF. (TIF) [file pcbi.1008525.s004.tif]

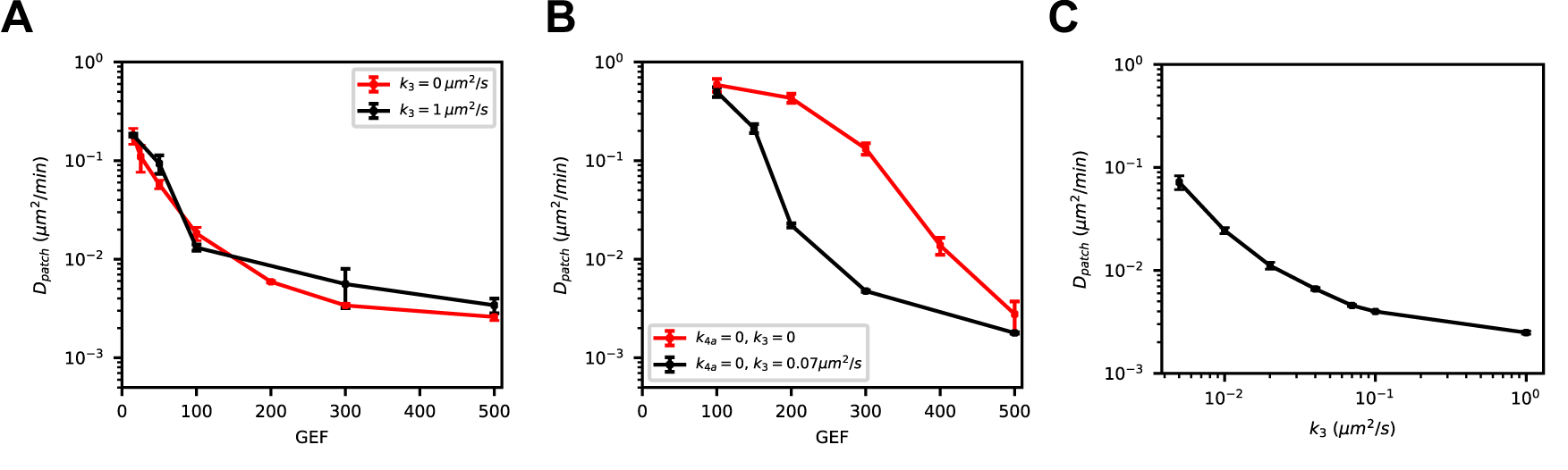

Supplement: S5 Fig — (A) Effective diffusivity of the patch (Dpatch) as a function of total available GEF in the updated model (including Reaction 7) for k3 = 0 and k3 = 1 μm2/s. (B) Similar to (A) except with k3 = 0 and k3 = 1 μm2/s keeping k4a = 0. (C) Effective diffusivity of the patch (Dpatch) as k3 is varied with 300 GEF molecules. Error bars are standard errors from the least-squared fit used to compute Dpatch. (TIF) [file pcbi.1008525.s005.tif]

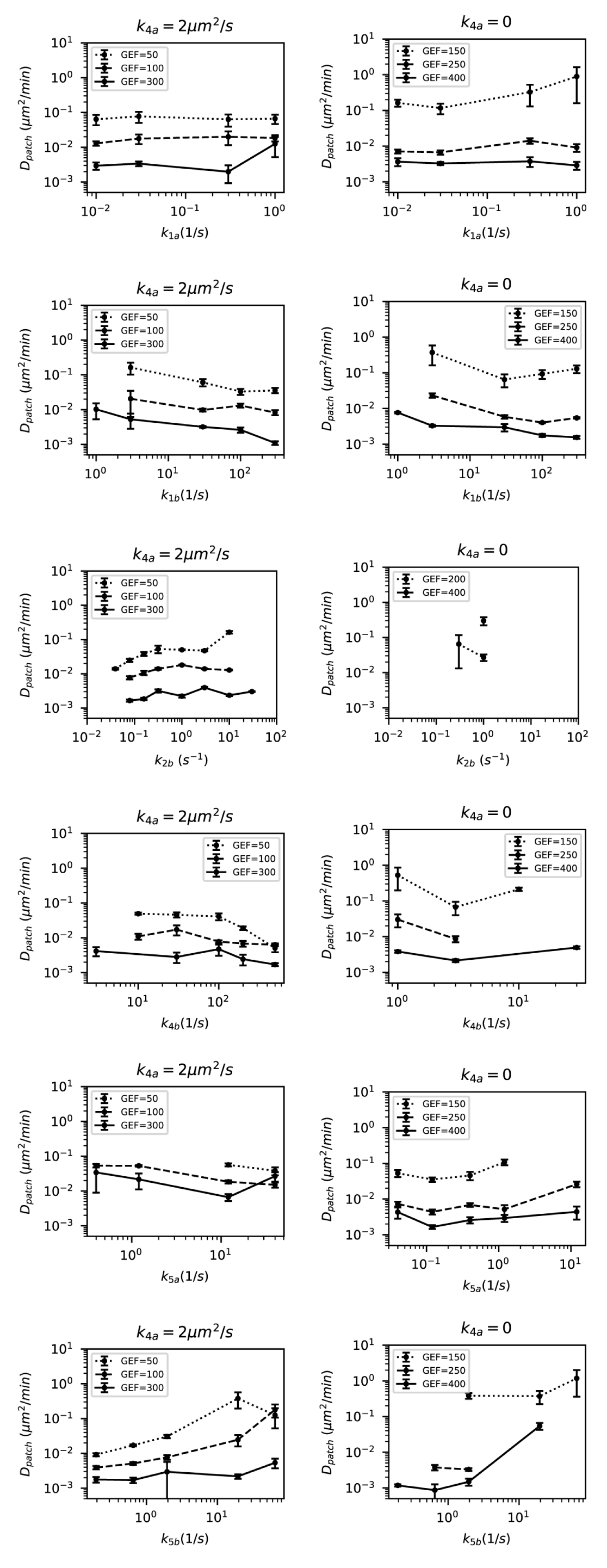

Supplement: S6 Fig — The panels on the left are for k4a = 2 μm2/s and for the ones on the right k4a = 0. Simulations were run with different GEF abundances as indicated. Missing points in each panel correspond to simulations that did not show robust polarization. Each data point was obtained from 5 simulations of 3600s each as described in the Methods. Error bars are standard errors from the least-squared fit used to compute Dpatch. (TIF) [file pcbi.1008525.s006.tif]
