# Supplementary material for: A novel stochastic simulation approach enables exploration of mechanisms for regulating polarity site movement
Source: PLoS Comput Biol. 2021 Jul 15;17(7):e1008525. doi: 10.1371/journal.pcbi.1008525 (PMC8315557; doi:10.1371/journal.pcbi.1008525)
Supplement: S1 Text — (DOCX) [file pcbi.1008525.s007.docx]

**S1 Text. Descriptions for S3, S5 and S6 Figs**

**Parameter exploration to enable polarization with low GEF numbers (S3 Fig)**

We varied different rate constants with goal of finding parameter sets that enable polarization with low GEF numbers. To evaluate if the distribution of active Cdc42 was polarized for a given parameter set we quantified clustering with H(*r* = 1.1 μm) (see Methods and Main Text). In most cases, a value of H(*r* = 1.1 μm) > 1.5 indicated there was polarization, but when the numbers of active Cdc42 are low the value of H can fluctuate substantially. Therefore, we also used the total amount of active Cdc42 as an additional criterion for polarization. For cases of highly variable clustering, amounts of active Cdc42 close to zero indicated no polarization.

Parameter changes that resulted in polarization with 100 GEF molecules or less were decreasing *k_2b_*, decreasing *k_4b_*, increasing *k_5a_* and increasing *k_7_*.

**Testing how different rate constants affect patch mobility (S5, S6 Figs)**

As described in the main text the parameter *k_4a_* has a strong influence on patch mobility. In our initial parameterization, the value of *k_4a_* is relatively high, making the patch stable and obscuring the effect of other parameters. For example, changing *k_3_* from 1 μm^2^/s to zero (in the updated model that includes Reaction 7) does not affect patch mobility significantly (S5 A Fig). However, when the value of *k_4a_* is set to zero, for a range of GEF abundances, changing *k_3_* from its original value of 0.07 μm^2^/s to zero increases patch mobility significantly (S5 B Fig). In S5 C Fig, for 300 GEF, we show that the change in patch mobility takes place gradually for different values of *k_3_*.

To test the effect of patch mobility of other rate constants, we started with the following parameterizations of the updated model (including Reaction 7) which show different levels of patch mobility:

- *k_4a_* = 2 μm^2^/s with GEF numbers of 50 (high mobility) 100 (medium mobility) and 300 (low mobility). These results are shown on the left column of S6 Fig.
- *k_4a_* = 0 with GEF numbers of 150 (high mobility) 250 (medium mobility) and 400 (low mobility). These results are shown on the right column of S6 Fig.

In S6 Fig each row corresponds to a particular rate constant tested as indicated on the x axis. We show data for all rate constants except for *k_2a_* and *k_4a_* which are presented in the Main Text and *k_3_* which is evaluated in S5 Fig.

Varying the value of most of the parameters did not substantially affect patch mobility. Modifying *k_5b­_* (the rate constant for Cdc42D to transition from the membrane to the cytosol) showed a moderate effect on patch mobility for *k_4a_* = 2 μm^2^/s. The effect was less robust when *k_4a_* = 0, in this case changing *k_5b­_* tended to destroy polarity (missing points in the curves).
