# Supplementary material for: A novel stochastic simulation approach enables exploration of mechanisms for regulating polarity site movement
Source: PLoS Comput Biol. 2021 Jul 15;17(7):e1008525. doi: 10.1371/journal.pcbi.1008525 (PMC8315557; doi:10.1371/journal.pcbi.1008525)
Supplement: Attachment — Submitted filename: response to reviewers.docx [file pcbi.1008525.s008.docx]

**Response to Reviewers**

We thank both reviewers for their constructive criticisms, which have significantly improved the manuscript. All sections where changes have been made are marked in red in the revised manuscript.

**Reviewer #1**: In this work, the authors explored the development of yeast cell polarization to an external pheromone gradient, investigating how the polarity factors formed clusters and then subsequently repositioned themselves before eventually forming a stable polarity site on the side of the pheromone source. This is entirely computational work, which builds on a model that the same group developed previously (McClure et al., Dev. Cell. 2015). The model is computationally intensive, so the authors developed new simulation methods that enable efficient simulation over an hour of real time; these new methods are a significant advance over existing ones. Intriguingly, they find that polarity cluster relocation does not necessarily rely on negative feedback, as had been posited previously, but can arise solely from stochastic noise.

Overall, this is very good work and appropriate for publication in PLOS Computational Biology. I have no concerns about any of the work presented, but I do have several suggestions for improvements, listed below. Note that I refer to several papers below, including from my own work, but am not requesting that the authors cite them; they may cite these papers if they wish but there is no pressure to do so.

We thank Steve for his positive opinion of work, his recommendation for publication and for his suggestions for improving the manuscript.

Major issues

I tried to download the code used in this work from <https://github.com/samuramirez/stochastic-exploratory-polarization>, as the manuscript describes, but the repository is empty. This clearly needs to be fixed.

The code has now been uploaded to the above github link.

The reaction rate terminology was somewhat confusing. To most biochemists, including most modelers, there is just some given reaction rate constant for a given reaction. It was initially unclear how this rate constant related to the microscopic rate, the mesoscopic rate, the scale-dependent mesoscopic rate, and the concentration-dependent mesoscopic rate. Are some of these synonyms for each other? Also, is there any physical meaning to the mesoscopic rate constants, or are they essentially just simulation parameters that cause the simulation to agree accurately with the mean association times?

We thank Steve for these comments and now appreciate that we did not clearly explain the connection between the various rate constants. To address this deficiency, we have significantly rewritten the Introduction and Background sections. Steve is correct that the mesoscopic rate constants are chosen using the criterion of preserving mean association times. They do have qualitative interpretations, based on the different assumptions used to derive them, which we note in the revised manuscript (6^th^ paragraph in the Introduction, second paragraph in Section A.1, Methods’ new subsection “Microscopic models for reacting particles”). We hope our revisions are sufficient to eliminate any confusion.

People who are familiar with Smoldyn know that its default simulation algorithm follows the Smoluchowski scheme, in which reactants only occur when two reactants are within a "binding radius" of each other and, if so, then react with probability 1. If the reaction is reversible, then Smoldyn separates the dissociation products by an "unbinding radius" that is typically larger than the binding radius. Also, Smoldyn has not been calibrated for quantitatively accurate reactions in 2D (see Johnson et al., BioRxiv 185595, 2020). However, this manuscript describes Smoldyn being used with Erban and Chapman's lambda-rho algorithm (Erban and Chapman, Phys. Biol. 6:046001, 2009) and having accurate 2D reactions, both of which are certainly possible with Smoldyn but not standard. I suggest checking what was really done and then clarifying this situation in the manuscript.

We used lambda-rho parameters within Smoldyn as the software allows such parameterization. The revised Methods now makes explicit how this was done in the section “2D particle-based simulations”.

On the same topic, the text is misleading in the description of particle-based simulation in lines 805-813. I don't believe that the lambda-rho model uses the linear approximation for the probability. Also, for first order reactions, Smoldyn does not use the linear approximation for the reaction probability but instead uses the exact equation. Finally, for unbinding, Smoldyn's default behavior is to call the location of the complex the "reaction location" and to then separate both dissociation products away from that location in opposite directions, using a random orientation, but with total separation equal to the unbinding radius (which is larger than the binding radius). It's possible that the authors set Smoldyn up to use a different method here but this should be checked.

Steve is correct that the lambda-rho model does not use a linear approximation. However, it does assume a single reaction rate when two reactants are within the capture radius. Therefore, for bimolecular reactions we set the reaction probability as *P* = Δ*t* λ, which is a valid approximation since for our parameters Δ*t* λ << 1. We make this point clear in the Methods section of the revised manuscript (subsection “2D particle-based simulations”).

We used Smoldyn’s standard routines for first order reactions and dissociation reactions. For the latter we specified the unbinding radius as r + e with e = 0.00001 to ensure molecules are not within the reactive distance the next time step. These points are clarified in the Methods section of the revised manuscript (subsection “2D particle-based simulations”).

Particle-based simulations are being treated here as the ground truth against which the other simulation methods are compared. Is there independent evidence that the particle-based simulations are actually correct in these situations?

Steve is correct that we are assuming that particle-bases simulations using the λ-ρ theory represent the ground truth. Of the simulation methods we consider, particle-based simulations most faithfully capture the underlying microscopic description of the system, because the only two assumptions are that reactions between two particles within the capture radius occur with a single kinetic step and the computational approximation of discretizing time. To ensure our choice of Δ*t* is not introducing significant numerical errors, we ran simulations with small values and verified that our results did not depend on the value of Δ*t* used. The revised Background (section A.1. first and second paragraphs) and the new Methods subsection “Microscopic models for reacting particles” now provide our rationale for taking particle-based simulation as the ground truth.

I suggest a little discussion about 2D reaction rate constants. They make good sense in PDE or RDME models but become a little less well defined in microscopic models. Are they actually meaningful in these contexts, and how should they be interpreted?

The issue with 2D systems is that, in general, macroscopic second-order rate constants are not well defined. It is possible to define them in the reaction-limited regime (well-stirred limit), but not in the diffusion-limited regime. We clarify these points in the revised manuscript (new Methods subsection “Microscopic models for reacting particles” third paragraph).

I was impressed by the similarity between the particle-based and lattice-based results presented in Figure 3. My experience is that it's relatively easy to get reaction rates to correspond, but it's harder to get spatial patterns to agree, and it can also be challenging to avoid lattice artifacts when using RDME-based models. See Andrews and Arkin, Current Biology, 16:R526, 2006.

We are happy to hear that Steve was impressed with the agreement of the spatial-Gillespie simulations with results from particle-based simulations. That was a major goal of this work. We attribute these results to the fact that our method allows relatively small grid elements to be used and the rate constants of the lattice models were derived to match behavior (mean association times) produced by microscopic models. We now cite the work of Andrews and Arkin to note the challenges of associated with lattice artifacts (section A.4 first paragraph).

Toward the end of section A.4, I appreciated the discussion of k_h vs. k_c. However, it wasn't clear what the conclusions were about which approach yields more accurate results, either in general or for specific situations, and how accurate those results are.

Based on this comment and the comment of Reviewer 2, we performed additional simulations to test the accuracy of the two methods (Sup Mat Fig 1). In particular, we fixed the number of reacting molecules in a system undergoing the reaction A+B → C and A+B ↔ C at A = B = 2000 and then ran simulations for different domain sizes. The results show that *k_c_* provides a good approximation for all the domain sizes tested, whereas *k_h_* loses accuracy as the density increases (smaller domains).

In general, for single reaction systems, *k_c_* performed better than *k_h_* for high concentrations and the methods produced comparable results for low concentrations. However, the polarity model contains multiple reactions and species in both high and low concentrations, so discerning which mesoscopic rate constant is more accurate is complex. We note however that using *k_c_* offers the possibility of using smaller lattice spacing than *k_h_* under diffusion-limited conditions, which in this study allows for higher accuracy (Figure 4 C,D), although at a higher computational cost. We also favor *k_c_* because it did not artificially induce polarization in parameter regimes where no polarization was observed in the particle-based simulations. In fairness, however, this method did not generate polarization for some parameters where particle-based simulations did.

We added a paragraph at the end of Section A.4. commenting on these points.

In section B.1, the authors explored the consequences of different GEF abundances. However, they didn't describe the current best guesses, or evidence, from the experimental literature, which would be useful to know.

Most simulation studies of yeast polarity have assumed GEF (Cdc24-Bem1) numbers in a range between 500 – 2000 molecules for a haploid yeast cell with a volume of ~48fL (Goryachev 2008, McClure 2015, Klunder 2013, Kuo 2014). Such studies, however, focused on regimes where the polarity patch has already been established and is stable. We believe that at early times following pheromone exposure when highly dynamic clusters are observed, the amount of effective GEF molecules that are available can be considerably smaller and may subsequently increase as GEF molecules are exported from the nucleus in a MAPK dependent manner. In this study, by varying the concentration of GEF molecules we are exploring the possible consequences of such regulation. This is now explicitly mentioned in Section B.1, second paragraph.

In the discussion section on "Highly dynamic clusters", the text mentions that the model does not include an explicit negative feedback mechanism. Is there nevertheless some sort of implicit negative feedback in the model?

There's no implicit negative feedback. Cluster dynamics are driven by intrinsic fluctuations.

Minor issues

Line 142 - Would read better with "... accurately represent... "

Modified.

Lines 144-147 - The reference to "scale-dependent rates" needs some explanation because most readers won't know what they are, at least at this point in the text.

We extended those sentences and provided more information.

Line 256 - Missing word "to".

Added.

Line 437 - Should replace "less" with "fewer".

Replaced.

Lines 815-817 - Do all membrane-bound molecules have the same diffusion coefficient? Also, it's worth pointing out that the rms displacement of a membrane-bound molecule is 0.1*rho, which is a very small step size (typically leading to slow simulations and high accuracy).

Yes, all membrane bound molecules have the same diffusion coefficient in the polarity models. The diffusion coefficients from the polarity models are however different from those of the elementary simulations presented in Fig 2. This is now clearly stated and commented in terms of the rms displacement in the Methods subsection “2D particle-based simulations”, 6^th^ and 7^th^ paragraphs.

Line 824 - Is there a reference for this equation?

This was replaced by Equation 2 in the revised manuscript in the new Methods subsection “Microscopic models for reacting particle”. This equation provides a bridge between the Smoluchowski and lambda-rho models by expressing and approximate relation between λ and the microscopic reaction rate *k_micro_*. In this way we can compare λ-ρ simulations with RDME simulations using scale-dependent rates that depend on *k_micro_*. The equation also assumes that the reactive distance ρ is the same for Smoluchowski and λ-ρ.

This is now clearly explained in the Methods subsection “Microscopic models for reacting particle”, 4^th^ paragraph.

Line 828 - The rho exponent didn't print correctly.

Fixed.

Lines 850-851 - Was the previous model deterministic or stochastic?

Deterministic. Added to the text. Methods subsection “Rate constants for the 2D stochastic polarization model” first paragraph.

Line 866 - The conversion between 2D and 3D certainly looks reasonable and yields the correct units, but is there a more rigorous justification for why it's correct?

It assumes that there are no cytosolic gradients perpendicular to the membrane due to fast cytosolic diffusion. We are assuming this is a good approximation since *D­_cyt_* >> *D_memb_*, but we have not evaluated when the approximation breaks.

Line 872 - I found the Th variable name to be confusing, especially since h is already a defined variable. How about delta-z, or something else that doesn't look like a product?

Th was replaced by Δz.

Line 941 - I wasn't familiar with the H(r) function or the Ripley's K function previously, and suspect that other readers might not know about them either, so I suggest slightly more explanation for them. Are they essentially just the integral of the radial distribution function, with a baseline offset?

We extended now the definition of the H(r), Ripley’s K function in a way that it is clear what is their relation to the pairwise-correlation function g(r).

For Table 1, I think it's worth pointing out that the 2D and 3D parameters are identical, except that a few cases required conversion due to dimensionality issues.

This is now explicitly mentioned in the caption of Table 1 and in the Methods subsection “3D particle-based simulations”.

Reviewed by Steve Andrews

**Reviewer #2**: In this paper the authors implement a stochastic simulation method that accounts for fluctuations due to molecular noise. The proposed mesoscopic method allows many particles to occupy a lattice grid, within which they react with a concentration-dependent rate. The method is then applied to the Cdc42 polarity patch movement, which exhibits diffusive mobility due to these molecular fluctuations. Overall, the work is well motivated, the work performed is extensive and results would be interesting to many researchers. However, the paper was difficult for me to read. I used most of my effort to understand the main assumptions of the proposed simulation method (which as I state below are not so clear to me). This subsequent detailed parameter-dependence of a complex polarization model, with multiple panels (e.g. Fig. 6 and 7) was then hard to follow and evaluate in the context of the new simulation method as well interpret the new results for the Cdc42 system.

We thank the reviewer for their overall positive opinion of our work. In hindsight, we agree that the paper was difficult to read. We extensively revised the manuscript with the goal of improving its readability.

Main comments:

1) The new method could be better placed in the context of extensive prior work on simulations of reacting systems. Terms such as “high/low densities” and “diffusion-limited” are used but these terms are not given very precise mathematical definitions to identify the different regimes in the system. So, it’s not clear to me if the cases of Fig. 2 are sufficient to validate the new method.

We added a new section “Microscopic models for reacting particles” to the Methods to include a more detailed discussion of prior work on reacting systems and diffusion limit.

To further validate our method, we have performed additional simulations that more systematically test the accuracy of the two mesoscopic methods under conditions of low and high densities (Sup Fig 1). In particular, we fixed the number of reacting molecules in a system undergoing the reaction A+B → C, A+B ↔ C at A = B = 2000 and then ran simulations for different domain sizes. The results show that *k_c_* provides a good approximation for all the domain sizes tested, whereas *k_h_* loses accuracy as the density increases (smaller domains).

To characterize the degree to which the system is “diffusion-limited” or “reaction-limited”, we used the dimensionless quantity λπρ^2^/*D*_tot_, where λ is the reaction rate used in the particle-based simulation, ρ is the reaction radius, D_tot_ is the sum of the diffusion coefficients of the reactants. For studying diffusion-limited reactions, we take λπρ^2^/*D*_tot_ = 50. This is now explicitly noted in the revised manuscript.

2) I think that the main assumption of the mesoscopic method, Eq. (14) needs to be clearly justified: is it rigorous and of not, what is the approximation introduced? This equation describes the reaction rate of molecules within the same grid. I see that in the reaction-controlled limit, the alpha*F term should be much smaller than one, and the reaction rate would obey law of mass action within the grid, as expected. When reactions start to be influenced by diffusion, then the alpha*F term that describes the resulting decrease in the reaction rate would start to become important. However, in this limit one would generally have a many-body reacting system within the grid, effectively described by time-dependent rate constants. Why is it ok to use a functional form for F that comes from a 2-particle calculation, over the grid diffusion time? Are any of the simulations in Fig. 2 and later in the limit where alpha*F is larger than one for it to matter?

We have attempted to better explain our rationale for using a concentration-dependent rate constant at the beginning of Section A.3 and in the Methods subsection “Mesoscopic concentration-dependent rate constant *k*_c_”.

The 2-particle calculation is justified because the effective area used in this calculation is related to mean free area of particles within the grid element. That is, when there are multiple particles in the same grid element the theory takes into account the fact that the area a particle has to “search” before encountering another particle is reduced. This effectively produces a time-dependent (and spatially dependent) rate constant, because the rate constant changes with the number of molecules of the more abundant species in each grid element.

In our new simulations (Sup Fig 1) the average value of alpha*F ranges from 0 - 4.25. These simulations demonstrate the accuracy of our approach for a range of alpha*F values.

3) I suggest that the authors simplify Figs. 5-9 to show the main conclusions with respect to mesoscopic method and any results that are important for the biology of the system. The analysis of the parameter dependence of the model is good, but I think it the readability will be greatly improved if the less important aspects are moved to an Appendix or Supplementary material.

We have attempted the streamline the second half of the paper “Section B. Mechanisms regulating mobility of the polarity site” to improve readability. We have also moved several of the results that previously appeared in Figs 6 to the Supplemental Material to better highlight key findings.

More minor comments:

4) Why are the simulations of Fig. 2A,B,E,F shown only at small times compared to the equilibration time?

We have doubled the simulation times to go out to 20s.

5) Why is the new method showing deviations that decrease with grid element size in Fig. 2F (line 297)?

The approximation we used for the dissociation mesoscopic rate *k_c_^d^* (Eq 19 in the revised manuscript) works better for smaller grid elements, we don't have a mathematical explanation for this. It is likely that the approximation works better for smaller number of molecules in the grid element, because *k_c_^d^* was derived using equations from a 2-body system.

6) How are the model parameters of Table 1 used in the different methods? Some comments in the table may help. For example, is the 3D model Smoldyn?

We now explain in the Table 1’s caption how to obtain parameters for the different methods. The revised Methods section also provides additional details. Both 2D and 3D particle-based simulations of polarity were performed in Smoldyn.

7) Lines 824, 828: the exponent for rho is missing

Fixed.
